# Source-Space Cross-Frequency Amplitude-Amplitude Coupling in Tinnitus

**DOI:** 10.1155/2015/489619

**Published:** 2015-11-19

**Authors:** Oliver Zobay, Peyman Adjamian

**Affiliations:** MRC Institute of Hearing Research, University Park, Nottingham NG7 2RD, UK

## Abstract

The thalamocortical dysrhythmia (TCD) model has been influential in the development of theoretical explanations for the neurological mechanisms of tinnitus. It asserts that thalamocortical oscillations lock a region in the auditory cortex into an ectopic slow-wave theta rhythm (4–8 Hz). The cortical area surrounding this region is hypothesized to generate abnormal gamma (>30 Hz) oscillations (“edge effect”) giving rise to the tinnitus percept. Consequently, the model predicts enhanced cross-frequency coherence in a broad range between theta and gamma. In this magnetoencephalography study involving tinnitus and control cohorts, we investigated this prediction. Using beamforming, cross-frequency amplitude-amplitude coupling (AAC) was computed within the auditory cortices for frequencies (*f*_1_, *f*_2_) between 2 and 80 Hz. We find the AAC signal to decompose into two distinct components at low (*f*_1_, *f*_2_ < 30 Hz) and high (*f*_1_, *f*_2_ > 30 Hz) frequencies, respectively. Studying the correlation of AAC with several key covariates (age, hearing level (HL), tinnitus handicap and duration, and HL at tinnitus frequency), we observe a statistically significant association between age and low-frequency AAC. Contrary to the TCD predictions, however, we do not find any indication of statistical differences in AAC between tinnitus and controls and thus no evidence for the predicted enhancement of cross-frequency coupling in tinnitus.

## 1. Introduction

Subjective tinnitus can be defined as an auditory phantom sensation in the absence of an external sound source and is only perceived by the person affected. The prevalence of tinnitus is estimated between 10 and 15% of the adult population [1], and although it is more common in the elderly (>60), it can appear at any age. In some cases, tinnitus can severely affect an individual's quality of life, with sleep deprivation, anxiety, and depression being the most common comorbidities of tinnitus [2].

The precise mechanism of tinnitus generation and maintenance remains elusive. While tinnitus is typically triggered by damage to the cochlea and the concomitant hearing loss, a large body of evidence from animal studies has revealed specific changes in neural activity at various structures of the auditory pathway, including the auditory cortex (for a comprehensive review see [3]). Reduced cochlear nerve activity and a subsequent reduction of activity within the affected peripheral auditory region are thought to downregulate inhibitory processes, which leads to hyperexcitability within central auditory structures, including primary auditory cortex [4]. In humans, early functional MRI studies implicated the involvement of both cortical and subcortical brain regions involving auditory as well as nonauditory structures ([5–8]; see also [9, 10] for reviews).

The thalamocortical dysrhythmia (TCD) model proposed by Llinás and coworkers [11, 12] presents a theoretical explanation for the underlying neurological mechanisms of tinnitus. The TCD model provides a general common framework for a range of neurological and neuropsychiatric disorders, such as Parkinson's disease, neurogenic pain, and depression. Applied to tinnitus, it asserts that thalamocortical oscillations lock a region in the auditory cortex into a slow-wave theta rhythm (4–8 Hz) and because of lateral disinhibition, the cortical area surrounding this region generates abnormal gamma (>30 Hz) oscillations (“edge effect”) which form the basis of the conscious tinnitus percept [11, 12]. Importantly, for electrophysiological studies of tinnitus, the model makes several specific predictions that are amenable to testing in magnetoencephalography (MEG) and electroencephalography (EEG) studies. First of all, TCD implies a number of changes in oscillatory power spectra, that is, a shift of normal alpha (8–13 Hz) oscillations towards low-frequency theta rhythmicity, and an increase in gamma and global power. A number of studies have investigated power changes in tinnitus [13–19], but the results so far remain somewhat inconclusive (for an overview, see, e.g., [20, 21]). While some studies were largely in agreement with TCD, others failed to see some of the predicted effects.

A further prediction of the TCD model concerns an enhanced cross-frequency coherence in a broad frequency range between theta and gamma, induced by the abnormal thalamocortical oscillation and the ensuing increased gamma activity [11, 12]. Electrophysiologically, the prediction can be investigated by analysing cross-frequency amplitude-amplitude coupling (AAC) [11], that is, correlations between signal amplitudes at two different frequencies *f*_1_ and *f*_2_ in the time-frequency spectrum. Empirical support for an increase in AAC has indeed been found in a number of studies and across various neurological conditions, both in comparison of patients to healthy controls (e.g., various conditions related to TCD [11, 22], schizotypy [23]) and in pre-post intervention studies (e.g., various conditions related to TCD treated by central lateral thalamotomy [24]; phantom limb pain treated by deep brain stimulation [25]). However, in a study of obsessive-compulsive disorder, significant coupling between the delta (1–3 Hz) and beta (13–30 Hz) bands as well as between theta and beta was found only for the controls but not the patients [26].

In tinnitus, indications for a reduction in cross-frequency correlations following transcranial direct-current stimulation (tDCS) have been reported [27], both within the dorsolateral prefrontal cortex (DLPFC) and between the DLPFC and a number of other brain regions. In a study of tinnitus and musical hallucinosis [28], significant correlations between low and high frequencies were found in the patient groups which were absent in the controls. However, a direct statistical comparison between patients and controls was not conducted.

Therefore, while these studies provide some empirical support for the increase in cross-frequency coherence predicted by TCD, the effect has not been demonstrated conclusively, and further investigations are necessary. This is particularly important given the known heterogeneity that exists between clinical characteristics in tinnitus participants [29].

The purpose of the present study was to test the predictions of the TCD model regarding cross-frequency power coherence in a cohort of tinnitus patients using resting-state MEG data. Using a beamformer approach to obtain source-space electrophysiological activity, our work focusses on studying cross-frequency coherence in the auditory cortices which are supposed to play a central role in the generation of the tinnitus percept. In contrast to previous studies, we examine frequency couplings over a wider frequency range (up to 80 Hz) since we are also interested in the effects in the high-gamma region. The TCD model as presented in [11, 12] predicts tinnitus-related changes in AAC in particular in two frequency windows (see [12, page 330]), that is, a low-frequency (theta) region (4 Hz ≤ *f*_1_, *f*_2_ ≤ 8 Hz) and, as a specific signature of the edge effect, a region connecting low and high (beta/gamma) frequencies (4 Hz ≤ *f*_1_ ≤ 8 Hz, 13 Hz ≤ *f*_2_ ≤ 40 Hz). However, in our main working hypothesis, we do not want to restrict the frequency location of potential AAC changes a priori, and we therefore conjecture that tinnitus can affect AAC at any frequency combination (*f*_1_, *f*_2_) within the investigated range (2–80 Hz). To test this assumption, we statistically compare frequency-resolved maps of AAC (comodulograms) by means of cluster-based permutation tests [30]. Nevertheless, as complementary hypotheses, we also directly investigate the specific TCD predictions and test AAC averaged over each of the two frequency windows described above. In further analyses, we study correlations between AAC and important covariates, such as age, hearing loss, tinnitus handicap, and tinnitus duration.

The present work continues our investigations of the TCD model based on the analysis of an MEG study, for which results on tinnitus-related spectral changes in a masker-silence paradigm [17] as well as on resting-state oscillatory power and functional and effective connectivity in tinnitus [21] have already been reported. The current work focusses on a different prediction of the TCD model and thus is complementary to these papers.

## 2. Methods

In this study, we reanalysed MEG recordings that were used for the connectivity analysis reported in [21].

### 2.1. Participants

The cohort consisted of the same participants as our earlier study [17] except for the addition of five controls and two tinnitus participants and the removal of four tinnitus subjects, based on the availability of resting-state data. Participants with hearing loss but no tinnitus were not considered as there were only 8 subjects in this group. The present study thus used the same subjects as [21]. Tinnitus participants had chronic subjective tinnitus for at least six months prior to recruitment and were recruited from the Nottingham Ear, Nose and Throat (ENT) Clinic, Nottingham Audiology Services, and NIHR Nottingham Hearing Biomedical Research Unit. Control participants were recruited from the general population. Anyone with pulsatile tinnitus, Ménière's disease, stapedectomy, and neurological disorders was excluded. All participants were strongly right-handed as assessed by the Edinburgh Handedness Inventory. The cohorts included 28 tinnitus participants (14 males, 14 females) with an average age of 54.7 years and 19 nontinnitus controls (10 males, 9 females) with an average age of 39.0 years. Ethical approval was granted by the Nottingham National Research Ethics Service (National Health Service) (Code number 08/H0408/89), and all participants gave written informed consent prior to enrolment.

Tinnitus severity was assessed using the Tinnitus Handicap Inventory (THI) [31], based on the following classification: slight (0–16), mild (18–36), moderate (38–56), severe (58–76), or catastrophic (78–100) [32]. Accordingly, 3 participants reported slight, 11 mild, 7 moderate, 5 severe, and 2 catastrophic tinnitus. Hyperacusis (hypersensitivity to sounds) was assessed using a standardised questionnaire where a score of >28 is indicative of hyperacusis [33]. Only 3 participants in the tinnitus group were classified with hyperacusis.  Table 1 summarizes the characteristics of the participants. The Tinnitus Tester software [34] was used to measure TI laterality, loudness, and dominant pitch. Further details of this procedure are provided in a previous paper [17, 35]. Pure-tone audiometry was collected for each participant for frequencies between 0.25 and 12 kHz. All nontinnitus controls had clinically normal hearing, which is defined as thresholds ≤ 20 dB between 250 Hz and 8 kHz, but in most cases we found increased thresholds at 12 kHz, indicating the existence of hearing loss at higher frequencies. Single-subject audiograms are shown in [21].

### 2.2. MEG Data Collection

MEG was recorded at a sampling rate of 600 Hz in a magnetically shielded room using a whole-head CTF system (VSM MedTech, Port Coquitlam, Canada), consisting of 275 radial gradiometers. Participants were lying in a supine position and head localisation was achieved by localising three electromagnetic coils attached to the nasion and left and right preauricular. Resting-state data consisting of alternating 1-minute eyes-open and eyes-closed segments were obtained with instructions introduced through an earpiece. MRI anatomical scans were obtained for each participant using a Philips 3T or 1.5T scanner. Images were T1-weighted rapid gradient echo sequence, with a matrix size of 256 × 256 × 256 and a defined voxel size of 1 × 1 × 1 mm^3^. Coregistration with the MEG data was performed using a surface-matching technique described in [36].

### 2.3. Data Preprocessing

MEG data were preprocessed and analysed in MATLAB (The MathWorks, Inc., Natick, MA) using the FieldTrip package (http://fieldtrip.fcdonders.nl/start) [37] and custom-written scripts. Raw data were bandpass filtered between 0.5 and 100 Hz and downsampled to 250 Hz. Further data analysis then proceeded with the eyes-open periods. This was to avoid the widespread alpha activity associated with eye-closure and drowsiness which can be misinterpreted as tinnitus-related activity. Segments of durations 5 s and 1 s were cut off at the beginning and end of each eyes-open period, respectively, to avoid any transients. A narrow bandstop filter was applied to remove line noise at 50 Hz. Independent component analysis was used to remove artifacts such as heart beat and eye blinks.

### 2.4. Source Analysis

Each subject MRI was individually labelled using the BrainSuite software (http://brainsuite.org/) [38]. The areas marked as superior temporal gyrus and Heschl's gyrus in each hemisphere were assumed to provide a good localisation of the subject's auditory cortices (ACs). After visually confirming that the BrainSuite labelling was accurate, 200 voxels were automatically selected in each hemisphere that were evenly spread out across the AC regions. Using the MRI-MEG coregistration, these voxels could then be localized in MEG headspace.

To compute time series for the virtual electrodes at the selected voxels, we used the linearly constrained minimum-variance (LCMV) beamformer with unit-noise-gain normalization [39–41] and the regularization parameter [42] set to 5% of the mean of the diagonal of the covariance matrix. Covariance matrices between sensor signals for use with the beamformer were computed separately for each segment of eye-open data.

### 2.5. Amplitude-Amplitude Coupling

AAC was computed following the method described in [43] which derives the couplings from the calculation of the power spectra. Each voxel time series was cut into segments of 2 s duration which were Fourier transformed using a Hanning window function. AAC between two frequencies *f*_1_ and *f*_2_ was then computed by correlating the squared moduli of the corresponding Fourier coefficients across segments. For our analysis, we considered frequencies between 2 and 80 Hz with a step size of 0.5 Hz.

To validate our numerical implementation of this approach, we also computed the AAC by filtering the source signal into narrow frequency bands (width 2 Hz) and correlating the squared amplitudes of the Hilbert-transformed filter outputs [44, 45]. We found very good agreement between the two methods, as expected on theoretical grounds. However, even though this second method might be more intuitive and is also closely related to the methodology for calculating other types of cross-frequency coupling [45], we find the spectrum-based approach [7] to be computationally faster by more than an order of magnitude and thus preferable in practical applications. For further confirmation, we calculated the couplings using a standard wavelet-based time-frequency analysis as described on the FieldTrip website. Again, we found this method to provide very similar results but to be less computationally efficient.

Computing couplings from a voxel time series for a matrix of frequency pairs (*f*_1_, *f*_2_) results in a comodulogram for this voxel. Averaging the comodulograms of all the voxels selected in the auditory cortices yields a subject's overall comodulogram. Taking the average of all couplings in a frequency region of this comodulogram provides the corresponding mean AAC (mAAC) as a more compact measure of coupling strength. The two frequency regions actually used for this purpose were directly related to specific predictions of the TCD model, as described in Introduction.

### 2.6. Correlations with Covariates

We computed frequency-resolved maps of the correlations between AAC and the main covariates characterizing our study sample. For each covariate, the corresponding map was obtained by calculating the across-subject correlations with AAC at all frequency pairs (*f*_1_, *f*_2_). The covariates comprised age, hearing level, THI sum score, tinnitus duration, and hearing level at the tinnitus frequency (with the latter three only including tinnitus subjects). Hearing level was computed as the mean audiogram level across all measured frequencies (250 Hz to 12 kHz).

### 2.7. Statistical Analysis

#### 2.7.1. Assessing the Presence of AAC

To investigate whether our data indeed showed evidence of AAC, comodulograms were computed after randomly permuting the time ordering of the Fourier coefficients for the 2 s slices, independently, for each frequency. These permutations break any existing correlation and thus AAC between frequencies (however, the same permutations were used across voxels to retain any spatial correlations). For each subject, 20 permutation comodulograms were computed in this way. Randomly drawing one of the comodulograms for each subject and averaging across controls or tinnitus participants provides a subject-averaged AAC-free comodulogram. Generating a large number of group averages in this way (*n* = 1000) results in a null distribution against which the observed averaged comodulograms can be compared. For further illustration, we also obtained comodulograms for synthetic white-noise sensor input.

#### 2.7.2. Frequency-Resolved Correlation Maps

Before testing for tinnitus-related effects on the comodulogram, we first examined the frequency-resolved correlations of AAC with age and hearing loss, respectively. These analyses were performed in order to assess whether these variables should be included as confounders when studying the effect of tinnitus on AAC. To protect against type I error inflation when testing the AAC-covariate correlations across all frequency pairs (*f*_1_, *f*_2_), we applied a cluster-based permutation test [30]. To this end, the correlation maps are thresholded at the significance limit for a corresponding univariate test (e.g., |*r*| ≥ 0.288 for a sample size of 47 at *α* = 0.05. Note that this thresholding rule only constitutes a convention; one could as well use larger or smaller threshold values resulting in more or less localized clusters). This yields a set of clusters, each of which is assigned the sum of the included absolute correlation values as the cluster statistic. One then creates a set of permutation correlation maps by randomly reshuffling the assignment of covariates to subjects. For each permutation map, the clusters are recomputed and the maximum cluster statistic is recorded. In this way, a null distribution of cluster statistics is obtained against which the observed statistics can be compared to derive *p* values. The correlation maps between AAC and the tinnitus-related covariates (THI, duration, and hearing level at tinnitus frequency) were analysed analogously after adjusting the thresholding limit to 0.374 (critical correlation for the sample size of the tinnitus group, *n* = 28).

#### 2.7.3. Comodulograms

The analysis of the correlation map provided evidence of an effect of age on AAC. We therefore included age as a confounder in the group comparison of the comodulograms between tinnitus and control subjects. This comparison was again based on a cluster-based permutation test. The mechanics of the test are analogous to those of the correlation test described above. The relevant statistic is now the partial correlation  *r*_AAC,Group|Age_ between AAC and group assignment controlling for age, which is computed for all frequency pairs (note that this partial correlation is simply a rescaled version of the group effect *β*_Tinnitus_ − *β*_Controls_ (see the appendix) and has exactly the same *p* value in univariate tests). The resulting map is thresholded at 0.291 (the significance limit of a partial correlation with one controlling variable at a sample size of 47). Computing the permutation samples for the partial correlations is somewhat more involved than in the case of the zero-order correlations described above; references and a brief outline of the methodology are given in the appendix. In order to assess how strongly the age covariate influenced the results and because we were not fully sure whether age should indeed be retained in the model (note that the inclusion of irrelevant variables reduces statistical power [46]), we repeated the analysis without including age. To this end, the standard *t* statistic was used as the test statistic (rather than the partial correlation), the threshold was set to 2.01, and the permutations reshuffled the group assignments between subjects.

#### 2.7.4. Hemispheric Comparison

In a further analysis, we considered the subsample with unilateral tinnitus (*n* = 17) and compared AAC between the auditory cortices ipsilateral and contralateral to the tinnitus. In this way, each subject acts as its own control. The comparison again made use of a cluster-based permutation test with the pairwise *t* statistic as test statistic, a threshold of 2.12, and permutations based on randomly assigning tinnitus laterality to the comodulograms in the left and right hemispheres.

#### 2.7.5. Analysing Mean AAC

As explained above, our main hypotheses and analyses considered frequency-resolved comodulograms and correlation maps. However, to test specific predictions of the TCD model regarding tinnitus-related changes in particular frequency regions of the comodulograms, we also examined AAC averaged over these regions as a more compact measure of cross-frequency coupling. As the distribution of this mean AAC (mAAC) across subjects appeared skewed and clearly non-Gaussian, but also for consistency with the frequency-resolved analyses, nonparametric permutation tests [47] were applied in all statistical work involving mAAC. Background and mechanics of these tests are briefly summarized in the appendix. The group comparison of mAAC between tinnitus and control subjects included age as a covariate, but we again conducted a complementary comparison disregarding age. To inform sample size calculations for future studies, we used the mAAC results to compute some estimates for the size of the tinnitus effect in terms of Cohen's *d* (note that, in the presence of the age covariate, Cohen's *d* is computed using equation (9.32) of [48]). Confidence intervals for *d* are obtained by bootstrapping using the R boot package [49–51].

## 3. Results

### 3.1. Single-Subject and Averaged Comodulograms

Comodulograms were computed between 2 and 80 Hz in steps of 0.5 Hz to obtain diagrams with high resolution. In all diagrams and analyses, we exclude frequency pairs with a difference of 1 Hz or less, as the corresponding AACs will be strongly affected by spectral leakage.

**Figure 1:**
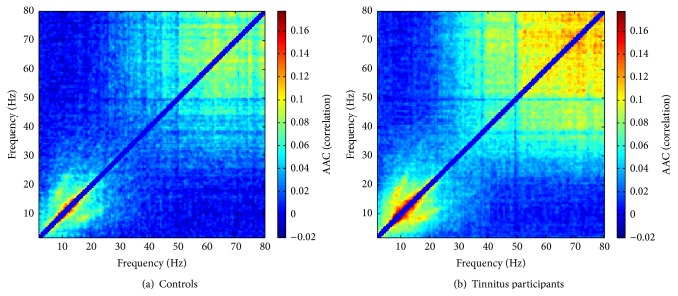
Subject-averaged AAC comodulograms for (a) controls and (b) tinnitus participants.

Single-subject comodulograms for all participants are shown in Supplementary Information (see Figure S1 in Supplementary Material available online at http://dx.doi.org/10.1155/2015/489619). As a general feature, clear manifestations of AAC are observed in the low-frequency region (LF-AAC, both frequencies below about 30 Hz) and/or the high-frequency region (HF-AAC, above about 30 Hz), but not for low-frequency/high-frequency combinations. For some subjects, both kinds of AAC are well pronounced, some display either LF-AAC or HF-AAC, and, for several, AAC seems largely absent. Nevertheless, this description should not be understood as a strict categorization; rather, there are smooth transitions between the different groups. In the averages over controls and tinnitus subjects, respectively, AAC is present at both low and high frequencies (Figure 1).

### 3.2. Establishing the Presence of AAC

**Figure 2:**
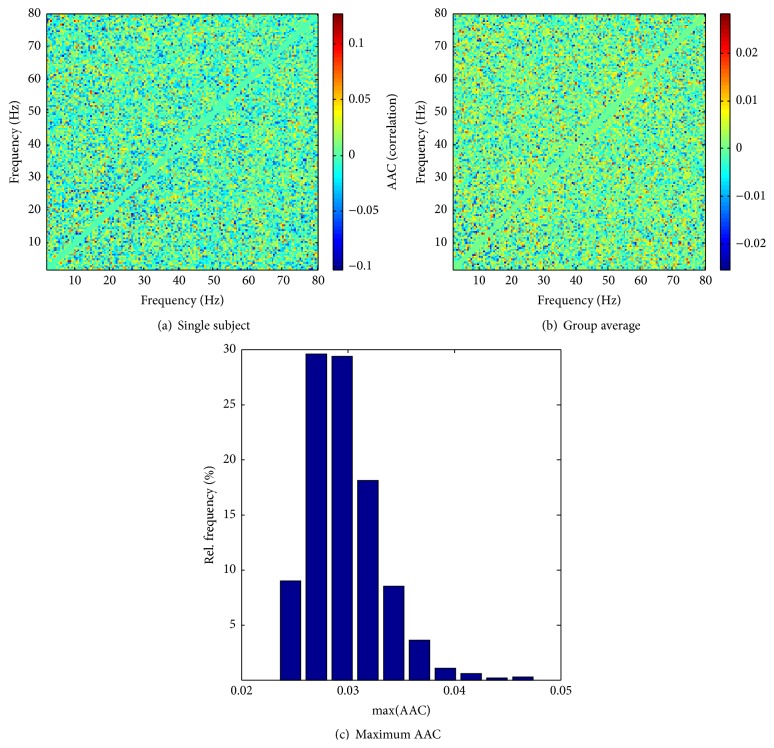
Comodulograms after removing AAC through reshuffling. (a) Single-subject comodulogram after averaging over voxels. (b) Average of single-subject comodulograms (as in (a)) over all controls. (c) Histogram of maximum AAC in control-averaged comodulograms for 1000 realizations.

The nonrandom patterns in the comodulograms clearly indicate that AAC is present in the electrophysiological signals; however, the existence of AAC can be more rigorously demonstrated by means of a statistical analysis. As described in Section 2, to this end, we computed a null distribution of subject-averaged comodulograms in the absence of AAC, using a shuffling algorithm.  Figure 2(a) shows a single-subject comodulogram computed in this way; Figure 2(b) gives an average over randomly drawn single-subject comodulograms across all controls (i.e., a member of the null distribution), and Figure 2(c) shows a histogram for the maxima of such averages over 1000 realizations. The upper limit of these maxima is at 0.047. As this is much smaller than the typical AAC values in the observed comodulograms (Figure 1), we can be sure that the observed patterns are not due to random sampling fluctuations in the absence of true AAC. This conclusion is corroborated by the fact that the comodulograms in Figure 2 do not show any discernible pattern. We also note that the observed AAC values, even for most of the individual subjects, are overwhelmingly positive, whereas the shuffling results contain a considerable fraction of negative correlations. We point out, as an aside, that the absolute values of AAC in the single-subject shuffling comodulograms are fairly large (Figure 2(a)) even though they are averages over 400 voxels. This is because the spatial correlations between voxels are retained in the calculations. This strongly reduces the “effective size” of the sample of voxels. However, averaging over subjects quickly reduces the mean AACs, in contrast to what is found for the actually observed AACs. We also computed AACs for white-noise input and obtained comodulograms that appear qualitatively similar to the shuffling results.

### 3.3. Correlation with Age and Hearing Loss

**Figure 3:**
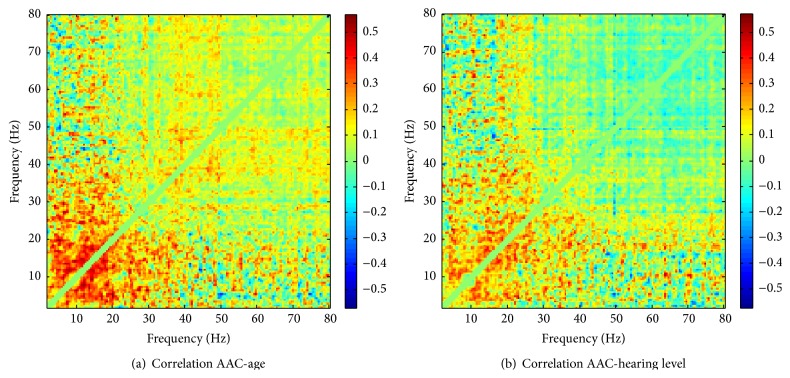
Frequency-resolved correlation maps between AAC and (a) age and (b) hearing level. The correlation at a frequency pair (*f*_1_, *f*_2_) is between the AAC values at that frequency combination and the respective covariate across all subjects.

As outlined in Section 2, we first investigated the correlations of AAC with age and hearing loss, respectively, to see if these variables should be included as confounders when testing for tinnitus-related effects on the comodulograms. The corresponding frequency-resolved correlation maps are shown in Figure 3. One sees that, for both covariates, correlations are most pronounced (and mostly positive) in the low-frequency regime (*f*_1_, *f*_2_ ≤ 30 Hz) whereas in the high-frequency regime they are largely absent. To illustrate the statistical testing of these maps, Supplementary Figure S2 shows the respective sets of clusters obtained from thresholding the maps at the significance limit of the corresponding univariate tests (|*r*| ≥ 0.29) as well as the clusters with the largest test statistic (i.e., sum of absolute correlations within clusters). A cluster-based permutation test with 2000 null samples assigned *p* values of 0.0435 (age) and 0.51 (hearing level) to these two maximum clusters. All other clusters were not significant. From these results, we conclude that there is evidence for statistically significant correlations between AAC and age in the low-frequency region between 10 and 20 Hz. However, we do not find evidence for a correlation between AAC and hearing level.

### 3.4. Comparing Comodulograms between Tinnitus and Control Subjects

**Figure 4:**
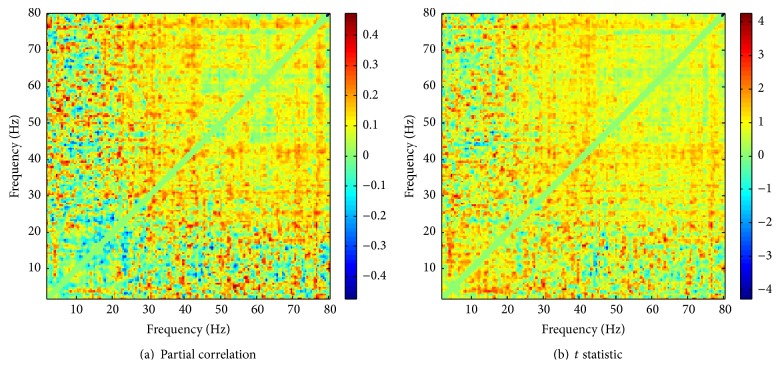
Frequency-resolved maps of (a) partial correlations between AAC and group assignment controlling for age and (b) *t* statistics for the group comparison between tinnitus and control subjects.

Based on the analysis of the frequency-resolved correlation maps of AAC with age and hearing loss, we decided to include age as a confounder in the test of tinnitus-related effects on the comodulograms. The relevant test statistic for this comparison is the partial correlation *r*_AAC,Group|Age_ between AAC and group assignment controlling for age.  Figure 4(a) shows the frequency-resolved map of these partial correlations, whereas the clusters obtained from thresholding the map are displayed in Supplementary Figure S3. These figures show that the partial correlations are weak and do not display any obvious pattern apart from, perhaps, a tendency towards positive correlations at higher frequencies. Clusters are very small and scattered. A cluster-based permutation test with 100 null samples yields a *p* value of 0.46 for the maximum cluster.

Even though the analysis of the correlation between age and AAC yielded evidence of an association, the result only just reached significance and the effect was limited to a region in the low-frequency regime. It is thus not completely clear whether age should indeed be included as a relevant confounder, and we therefore repeated the comodulogram comparison disregarding age effects.  Figure 4(b) shows a map of the two-sample *t* statistic obtained from the AAC data of tinnitus subjects and controls, and Figure S3(B) displays the observed clusters obtained by thresholding this map. These plots again do not suggest any systematic differences between the two groups, and the permutation test yields a *p* value of 0.37 for the maximum cluster (however, we note that the *t* statistic is positive for 90.2% of all frequency pairs between 2 and 80 Hz).

From these results, we conclude that our data do not show any evidence of tinnitus on cross-frequency amplitude-amplitude couplings, independent of whether age effects are taken into consideration.

### 3.5. Hemispheric Comparison

**Figure 5:**
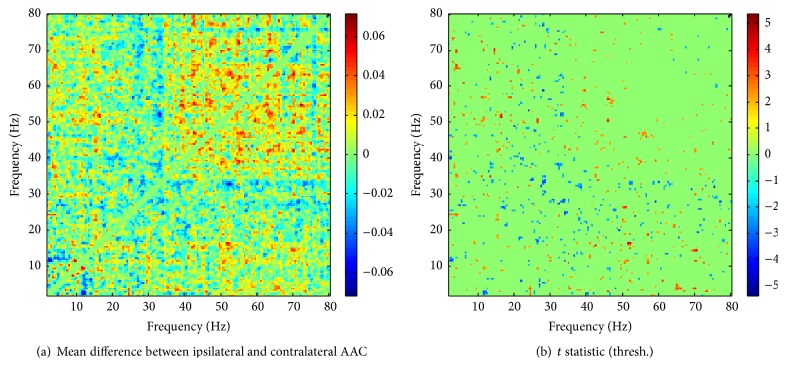
(a) Mean difference in AAC between the auditory cortices ipsilateral and contralateral to the tinnitus, in subjects with unilateral tinnitus. (b) Thresholded map of the corresponding pairwise *t* statistic showing the observed clusters used in the permutation test.

As an alternative to searching for an effect of tinnitus on AAC by comparing tinnitus subjects and controls, we also considered the subsample with unilateral tinnitus (*n* = 17) and tested for differences in AAC between the auditory cortices ipsilateral and contralateral to the tinnitus. In this way, each subject acts as its own control, thus reducing the effects of any potential confounders. The rationale of this approach is that tinnitus might affect the two hemispheres differently, depending on laterality.  Figure 5(a) shows a map of the mean differences in AAC between the ipsilateral and contralateral auditory cortices, and Figure 5(b) depicts the clusters obtained from thresholding the pairwise *t* statistic. These figures do not give any indication of a systematic difference in AAC between the auditory cortices, and this conclusion is confirmed by the result of a cluster-based permutation test which yields a *p* value of 0.67 for the largest cluster.

### 3.6. Correlation with Tinnitus-Related Covariates

**Figure 6:**
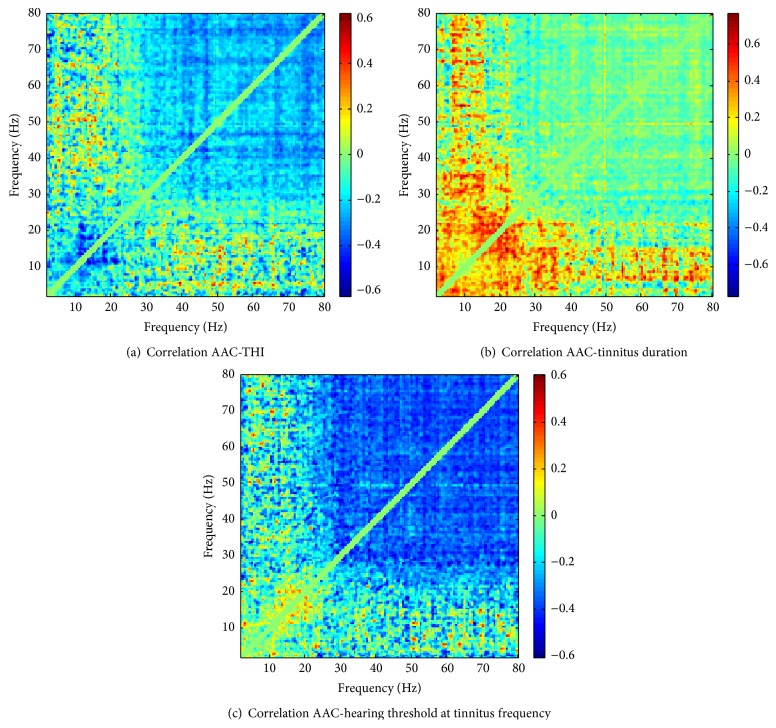
Frequency-resolved correlation maps between AAC and (a) the THI score, (b) tinnitus duration, and (c) the hearing threshold at the tinnitus frequency.

In a set of further analyses, we investigated correlations of AAC with a number of covariates only measured for tinnitus subjects, that is, the Tinnitus Handicap Inventory (THI) score, tinnitus duration, and the hearing threshold at the tinnitus frequency. Figures 6 and S4 show the corresponding correlation maps, the cluster sets, and the maximum clusters. The maps show some overall structure, for example, predominantly positive correlations with tinnitus duration in the low-frequency regime and an extended area of negative correlations with the hearing level at the tinnitus frequency in the high-frequency regime. However, the permutation tests do not provide clear evidence of significant associations; the *p* values for the three maximum clusters shown in Figures S4(D)–S4(F) are 0.191, 0.090, and 0.0515 for permutation tests with 2000 null samples.

### 3.7. Analyses for Frequency Windows Suggested by Llinás et al. [11, 12]

Complementary to the analyses described above that do not a priori single out any specific frequency regions, we have also investigated AAC in two smaller specific frequency windows (see Figure S5). These were suggested by Llinás et al. [11, 12] to be particularly sensitive to the effects of the neurological disorders described by the TCD model. The first window is in the theta-frequency region (4 Hz ≤ *f*_1_, *f*_2_ ≤ 8 Hz) and is supposed to directly reflect effects of the slowed thalamocortical dynamics. The second window is between the theta and the beta and gamma bands (4 Hz ≤ *f*_1_ ≤ 8 Hz, 13 Hz ≤ *f*_2_ ≤ 40 Hz) and relates to the edge effect. For each of these windows, we computed the mean amplitude-amplitude couplings (mAAC) as a more compact measure of cross-frequency coherence. The results of our analyses are summarized in Table 2. They confirm the conclusions from the frequency-resolved analyses. There is an indication of a positive association between mAAC and age; the correlation is significant in the theta-beta/gamma window and close to significance in the theta window. As a main result, however, we find that also for these specific frequency windows there is no evidence of a tinnitus-related effect on the couplings, with or without including age as a covariate. For the theta-beta/gamma window, the group comparison is almost significant before age correction but does not remain so when age is included. The results for the effect size (Cohen's *d*) show that the point estimates are mostly small, but there is still considerable uncertainty as the widths of the confidence intervals are always larger than 1. Finally, the hemispheric comparisons for unilateral tinnitus also do not give any indication of differences in mAAC between the ipsilateral and contralateral auditory cortices.

## 4. Discussion

Motivated by the predictions of the TCD model and recent reports in the literature, in this study, we examined potential effects of tinnitus on amplitude-amplitude cross-frequency coupling in the auditory cortices using MEG resting-state data. Our analyses did not find any statistically significant evidence of such an effect.

To affirm the TCD model, a previous MEG study by Llinás et al. [11, 12] used power correlation analysis to compare a set of nine subjects with various neurological disorders to an equally sized sample of healthy controls. They reported a difference between the group averages across all frequencies from theta to gamma but did not conduct any statistical analysis. Our study uses a considerably larger group of participants (28 tinnitus and 19 control subjects), focusses on a single disorder, and statistically analyses cross-frequency power correlations in a larger frequency range including high gamma. Current evidence is leaning towards the engagement of nonauditory brain areas in tinnitus perception and maintenance. As our analysis concentrated on the prediction of the TCD model and the auditory cortex in particular, it is possible that significant differences exist in other brain regions yet to be determined. We hope to perform and report this analysis in a future study. The present work serves to illustrate the feasibility of investigating AAC in tinnitus with the methods employed.

An important question in the interpretation of our results concerns the role of potential confounding variables. The original design of the MEG study considered four groups of subjects, that is, participants with and without tinnitus and/or hearing loss [17]. For the work of [21] as well as the present investigation, we decided to pool the tinnitus subjects based on our previous conclusion that a distinction between tinnitus with and without hearing loss is probably not meaningful [17, 52]. As an important consequence, the power of the comparisons between the tinnitus and control groups is increased due to the concomitant increase in sample size of the tinnitus group. However, merging the two tinnitus groups also leads to a mismatch in age between the controls and the new tinnitus group (39.0 versus 54.7 years). This means that the comparisons might potentially be confounded by the effects of age. Another potential confounder could be given by the differences in hearing level.

As to the latter, we decided not to control for hearing loss in our analysis based on our observation that there is no evidence for a correlation between AAC and hearing level. This implies that, strictly speaking, our analysis does not investigate the pure effect of tinnitus but rather the combined influence of tinnitus and hearing loss (most tinnitus subjects have hearing loss while all control subjects possess normal hearing). We do not find any evidence of such an influence and thus conclude that this combined effect most likely is small, if it exists at all. Our interpretation of this result is that the separate effects of tinnitus and hearing loss are also small. In principle, it would be conceivable that the two separate effects are larger but of opposite magnitude so as to cancel each other out. However, this seems unlikely, given that we do not observe an association between AAC and hearing level.

As to age, evidence for a correlation with AAC was found in the frequency-resolved correlation map as well as in the study of mAAC (Table 2). In the analysis of tinnitus-related effects on AAC, we therefore decided to correct for confounding effects of age by means of ANCOVA-like regression adjustments. We believe that this approach is justified for two reasons. First of all, even though the two groups are not well matched in mean age, the respective age distributions still overlap well; that is, both groups contain subjects at the lower and upper ends of the overall age distribution (age range: controls 23–64 years, tinnitus 24–70 years). We are thus* not* comparing a young to an old group for which ANCOVA would not be appropriate as it would involve extrapolation. Secondly, using cluster-based permutation testing, we have compared the ANCOVA model (in which both groups have the same slope coefficient for the age covariate, i.e., no group-age interaction) to a more general model where the slopes for age can differ with group (group-age interaction). As we did not find any evidence for a group-age interaction, we believe that the ANCOVA model is reasonable for our data.

Even though we found a significant association between age and AAC, this was limited to the low frequencies (see Figure S2(C)) and the evidence was not very strong (*p* = 0.044). We are therefore not completely sure whether it is indeed necessary to include age as a relevant confounder. Given that the inclusion of irrelevant variables leads to a loss in power to detect the effects of interest [46], we decided to repeat the analyses without the age confounder in order to assess the sensitivity of the results with regard to the effects of age. Both approaches, with and without the age confounder, consistently find no evidence of any effect of tinnitus on AAC which we therefore consider to be a robust conclusion from our data. The group differences apparent in the visual comparison of the comodulograms in Figure 1 are thus either due to age effects (i.e., more pronounced AAC in the tinnitus group which has a higher mean age) or, simply, due to sampling fluctuations.

In addition to the frequency-resolved analysis of AAC, we considered also mean AAC in two specific frequency windows described by Llinás et al. [11, 12] (see Figure S5). Our results for these windows (i.e., effect of age but not of tinnitus) were consistent with the findings from the frequency-resolved analysis. The hemispheric comparison within the subsample of subjects with unilateral tinnitus also did not show any evidence of a difference in mAAC between the auditory cortices ipsilateral and contralateral to the tinnitus. As each subject acts as their own control, this comparison is less susceptible to confounding effects, but it hypothesizes a measurable difference in the neuronal activity of the two hemispheres in unilateral tinnitus. It is possible that this assumption does not hold.

Nevertheless, from this absence of evidence, we cannot conclude that an effect does not exist at all in the auditory cortices; it is still possible that a larger sample (ideally with improved matching between subject groups) might reveal differences in AAC. The various point estimates for the effect size (Cohen's *d*) shown in Table 2 vary between −0.11 and 0.54, with a considerably larger range covered by the confidence intervals. If, for the sake of illustration, we assumed a true effect size of, say, 0.25, a test with 80% power would require a sample size of about 200 per group. Such a study is certainly not easily feasible, but we note that the analysis of large tinnitus-related MEG/EEG datasets may potentially soon become possible through the recent European Cooperation in Science and Technology (COST) Tinnitus Research Network (TINNET, http://tinnet.tinnitusresearch.net/index.php/the-action/tinnet-cost-action-bm1306.html). The goal of this COST action is to identify subtypes of tinnitus and to better understand the heterogeneity of tinnitus with the ultimate aim of identifying suitable treatments for the condition. A part of the action involves creating a large database of MEG/EEG data of potentially hundreds of participants from different European research centres. Given the significance of the TCD model for the theoretical modelling of tinnitus, we hope that larger-scale studies of AAC will be carried out in the future once the database has become available.

In contrast to other forms of MEG analyses, for example, connectivity studies, source leakage does not pose a particular problem in the present investigation. In connectivity analysis, the objective is to assess the similarity between the time series at two voxels A and B. If the signals at both of these locations are strongly affected by a source at location C due to field spread, then it is possible to detect a spurious connectivity between A and B [53]. In contrast, AAC describes the similarity between the amplitude time series of different frequency components of the signal at a* single* voxel. In the presence of source leakage, AAC measured at a voxel A might be contaminated by the AAC of source C, but it is not possible to generate a completely artifactual result as could happen in connectivity analysis. In other words, we are measuring real AAC, but with limited spatial resolution.

Limited resolution is a problem of all source-space analysis techniques and hence for any variable recorded in source space. In addition, however, our analysis also averages over the auditory cortices, thus further reducing the spatial resolution. Is it possible that our failure to detect the effects on AAC predicted by the TCD model is due to a lack of resolution? We cannot exclude this possibility, but according to the TCD model and the analyses of Llinás and coworkers it does not seem likely. Reference [11] asserts that the abnormal low-frequency activity will not remain restricted to a small region but through the entrainment of the nonspecific system result in “the promotion of large-scale, low-frequency oscillatory coherence.” In fact, in their MEG analysis, the authors observed “an abnormal distribution and coherence of low-frequency activity over wide areas of the brain.” It is thus clear that in the TCD model high spatial resolution is not a prerequisite for observing effects on AAC. However, some brain regions are more strongly affected than others, such as the auditory cortices in tinnitus [12]. Overall, our strategy of averaging AAC over the auditory cortices therefore seems well suited to investigate the TCD predictions.

It is interesting to compare the comodulograms obtained in this work to the results of other recent studies [11, 22–25, 28] which also assessed cross-frequency coherence and AAC in various neurological conditions related to TCD. A detailed comparison is difficult due to numerous differences between studies such as data collection and the methodologies for computing couplings. Nevertheless, we find the relatively close visual similarity of our results to the findings of Llinás and coworkers [11] (Figure 4 in their work) quite remarkable. While other published comodulograms also show comparable features, we note more pronounced differences to similar EEG studies [23, 28]. In these cases, subject-averaged AACs take on rather large values (0.6 and above) even far away from the diagonal of the comodulogram (i.e., for large differences within the frequency pairs), and there appears to be a distinction in the behaviour of frequencies below and above 15 Hz, approximately. It needs to be determined whether these differences are due to differences between MEG and EEG [13] or some other reason.

Associations between behavioural measures of tinnitus and electrophysiological markers have repeatedly been reported before [54–56]. In the present study, we do not find evidence for correlations of AAC with the THI score and tinnitus duration, but we observe a negative correlation with the hearing level at the tinnitus frequency which almost reaches significance at high coupling frequencies (*p* = 0.052; see Figure S4(F)). However, this observation is not easily reconcilable with the TCD model. Assuming an overall positive association of tinnitus with such hearing loss on the one hand and with AAC on the other, as stipulated by the TCD model, one would expect this correlation to be positive as well.

Our work also gives rise to a number of open questions. First, it is necessary to clarify the origin and mechanism of AAC as such. In the case of phase-amplitude coupling (PAC) which lately has received much wider attention than AAC [57] there seems to be a consensus that PAC serves to bind together low- and high-frequency brain rhythms. The origin and significance of AAC do not seem to have been discussed in detail yet, but at least one particular way of generating AAC is readily conceivable. The time courses of well-defined oscillatory brain rhythms are often nonsinusoidal (see, e.g., [58], Figure 1, for the theta rhythm); that is, they contain a series of higher harmonics. AAC then simply results from the Fourier analysis of such a nonsinusoidal brain rhythm whose overall amplitude fluctuates in time. This idea is corroborated by the observation that in the averaged comodulograms of Figure 1 there is a clearly discernible off-diagonal maximum of AAC on the line *f*_2_ = 2*∗f*_1_ (or, equivalently, *f*_1_ = 2*∗f*_2_) in the low-frequency region which in our interpretation corresponds to the contribution of the first harmonic. However, this effect is much weaker for single-subject comodulograms, and a corresponding behaviour is not seen at all in the high-frequency region. It is therefore likely that other mechanisms are also at play in bringing about AAC.

A second, closely related issue concerns a better understanding of the separation of the AAC comodulograms into a low-frequency and a high-frequency component. We noted above that the comodulograms of [23, 28] obtained with EEG have a somewhat different appearance, so it is possible that the modality of data acquisition may play some role in this regard. We also note that there are pronounced qualitative differences between the comodulograms for individual subjects, as shown in Figure S1. On the one hand, these differences strongly suggest that the observed AAC is indeed of biological origin and not due to some unknown technical artefacts (unfortunately, empty-room data was not collected in this study). On the other hand, it is clearly necessary to better understand the cause of these differences.

Finally, we point out that in this study we concentrated on AAC in the auditory cortices. This is because of the assumptions of the TCD model according to which tinnitus is caused by an edge effect in the auditory cortex, which gives rise to increased cross-frequency coherence. It is therefore natural to assume that this coupling is strongly pronounced in the auditory cortex. However, it is now believed that tinnitus involves a distributed cortical network involving other brain regions [59, 60]. It is therefore necessary to extend the current investigation and thoroughly explore tinnitus-related effects on AAC in other regions of interest, and we hope to carry out work in this direction.

In conclusion, in this study we examined a specific prediction of the TCD model which claims the existence of increased cross-frequency amplitude-amplitude coupling in tinnitus. We did not find any statistically significant evidence of this effect, and our analyses suggest that if there is an influence of tinnitus at all, the corresponding effect size will likely be small. Nevertheless, because of the importance of testing the TCD model and given the expected creation of neuroimaging data bases such as TINNET, we hope that more powerful large-sample studies will be carried out in the near future. Our current analyses provided evidence for a correlation between age and AAC, and future work should therefore take potential confounding effects of age into consideration in the study design. We did not find any association between AAC and tinnitus-related covariates such as the THI scores, but these questions could also be readdressed in a larger-scale study. Such work might significantly contribute to our understanding of how AAC is generated neurophysiologically and how it is affected by tinnitus.

## Supplementary Material

The Supplementary Material contains additional figures supporting the analyses described in the paper, e.g., individual comodulograms for all participants, thresholded comodulograms, and diagrams of the clusters with maximum cluster statistics.

## Figures and Tables

**Table 1:** Participant characteristics. SD: standard deviation. Note that audiogram data are missing for two control subjects.

		Tinnitus	No tinnitus	
Gender	Male	14	10	*χ* ^2^(1) = 0.031
	Female	14	9	*p* = 0.90

Age	Mean (SD)	54.7 (12.8)	39.0 (14.0)	*t*(45) = 3.96 *p* < 0.001

Pure-tone average (0.25–8 kHz)	Mean left (SD)	25.2 (18.5)	4.5 (6.4)	*t*(43) = 4.43 *p* < 0.001
	Mean right (SD)	21.1 (16.5)	4.0 (5.7)	*t*(43) = 4.11 *p* < 0.001

Tinnitus Handicap Inventory (THI)	Mean (score) (SD)	39.9 (21.1)	NA	

Hyperacusis	*N*	3	NA	
	Mean (score) (SD)	17.0 (9.4)	NA	

TI quality	Tonal	19	NA	
	Hissing	3	NA	
	Ringing	6	NA	

TI laterality	Right	6	NA	
	Left	11	NA	
	Bilateral	11	NA	

TI duration (years)	Mean (SD)	12.9 (15.1)	NA	

**Table 2:** Summary of analyses for specific frequency windows described by Llinás et al. [11, 12]. *r*_mAAC,Group∣Age_ denotes the partial correlation between mAAC and group assignment controlling for age. A positive correlation indicates an increase of mAAC in the tinnitus group compared to the controls. *r*_mAAC,Age_ is the zero-order correlation between mAAC and age. *t*_mAAC~Group_ gives the two-sample *t* statistic for comparing mAAC between tinnitus participants and controls. All effect sizes are measured in terms of Cohen's *d*; CI denotes the confidence interval for *d*. The hemispheric comparison is between mAAC in the auditory cortices ipsilateral and contralateral to the tinnitus in subjects with unilateral tinnitus; the *t* statistic is for a pairwise test. All *p* values are computed by means of nonparametric permutation tests.

	Theta window (4 Hz ≤ *f*_1_, *f*_2_ ≤ 8 Hz)	Theta-beta/gamma window (4 Hz ≤ *f*_1_ ≤ 8 Hz, 13 Hz ≤ *f*_2_ ≤ 40 Hz)
*r*_mAAC,Group∣Age_	−0.048 (*p* = 0.75)	0.082 (*p* = 0.58)
Effect size *d*	−0.110 (CI: −0.84, 0.70)	0.188 (CI: −0.47, 0.89)
*t*_mAAC~Group_	0.635 (*p* = 0.54)	1.808 (*p* = 0.070)
Effect size *d*	0.189 (CI: −0.48, 0.81)	0.537 (CI: −0.03, 1.10)
*r*_mAAC,Age_	0.264 (*p* = 0.07)	0.384 (*p* = 0.004)
Hemispheric comparison	*t* = −0.418 (*p* = 0.68)	*t* = −0.122 (*p* = 0.91)

## Appendix

### Permutation Tests

Null-hypothesis significance testing requires the knowledge of the sampling distribution of the test statistic under the null. This knowledge allows computing the *p* value, that is, the probability of obtaining a statistic under the null that is at least as extreme as the actually observed one. If we can describe the data generation process by a parametric model, the sampling distribution can often be derived analytically, at least to a good degree of approximation. However, in cases where parametric assumptions cannot be made, nonparametric permutation testing may provide an alternative way of determining the sampling distribution.

As a simple example, consider the null hypothesis that a variable (e.g., mAAC) has the same probability distribution in two populations (e.g., tinnitus subjects and controls). As we suspect that the two populations have different means, we use the *t* statistic as test statistic, but other statistics could be used as well and may be more powerful for particular types of deviations from the null. Now assume that we observed the sample *Y* = (*y*_1_,…, *y*_*N*_) with the first M observation for tinnitus subjects and the subsequent N-M ones for controls. Under the null, however, the group memberships are essentially immaterial since by assumption there is no difference between the two groups with regard to the mAAC distribution. It therefore appears intuitively plausible that under the null the same sample *Y* could have been observed with equal probability if the first N-M subjects had been controls and the rest tinnitus subjects or, in fact, for any other permutation of group memberships. We can therefore approximate the sampling distribution of the *t* statistic under the null by drawing a large number of random permutations of group membership and computing the corresponding *t* values from the observed *Y*. The two-sided *p* value then equals the fraction of draws for which |*t*| ≥ |*t*_obs_| with *t*_obs_ being the observed *t* statistic. A rejection of the null implies that the mAAC distributions differ in some way between the two groups, probably, but not necessarily, including a difference in means.

As a further basic example, consider the case of paired observations ((*x*_1_, *y*_1_),…, (*x*_*n*_, *y*_*n*_)), a null hypothesis of independence between the random variables *x* and *y*, and their correlation *r* as test statistic. Under the null, any permuted sample ((*x*_1_, *y*_*π*(1)_),…, (*x*_*n*_, *y*_*π*(*n*)_)), with *π* being a permutation of integers 1 to *n*, has the same probability to appear. The sampling distribution of the test statistics under the null thus is computed by drawing a large number of permuted samples and computing the corresponding correlations. The two-sided *p* value then equals the fraction of draws for which |*r*| ≥ |*r*_obs_|. The rejection of the null implies an association, and most likely a nonzero correlation, between the two variables.

For the group comparisons of mAAC between tinnitus and control subjects, we need to include age as a covariate. Similar to a standard parametric ANCOVA, these comparisons model the dependence of mAAC on group membership and age as mAAC = *β*_Group_ + *β*_Age_ *∗*Age + *ε* and test for a difference in *β*_Group_ between the tinnitus and control groups. However, contrary to the ANCOVA, it is no longer assumed that the random contributions *ε* follow a normal distribution so that we have to apply a nonparametric test. As explained in [61], an exact permutation test of *β*_Tinnitus_ = *β*_Control_ ( = *β*_0_) is not available, and we follow their recommendation to use an approximate method developed by Freedman and Lane [62]. The null hypothesis is mAAC = *β*_0_ + *β*_Age_ *∗*Age + *ε*, and the fact that *β*_Age_ is not known precludes an exact test. The test statistic is the partial correlation *r*_mAAC,Group|Age_, and its sampling distribution under the null is computed with the help of pseudoobservations mAAC^*∗*^ obtained from permuting the residuals of the regression of mAAC against age. For details, see [61].

For the group comparisons of the frequency-resolved comodulograms in the presence of the age covariate, we also applied the algorithm of [62] to compute the permutation samples of the partial correlations. In this case, one has to ensure that in the calculation of a particular null comodulogram the same permutation pattern of residuals is used across all frequency pairs in order to preserve the correlations between the AAC values.
