# Supplementary material for: Source-Space Cross-Frequency Amplitude-Amplitude Coupling in Tinnitus
Source: Biomed Res Int. 2015 Nov 19;2015:489619. doi: 10.1155/2015/489619 (PMC4668294; doi:10.1155/2015/489619)
Supplement: Supplementary file 1 — The Supplementary Material contains additional figures supporting the analyses described in the paper, e.g., individual comodulograms for all participants, thresholded comodulograms, and diagrams of the clusters with maximum cluster statistics. [file 489619.f1.pdf]

Supplementary figures

Figure S1. The figures below show single-subject comodulograms of amplitude-amplitude cross-frequency coupling (AAC) in the auditory cortices for the frequency regime between 2 and 80 Hz. As indicated by the subject identifiers in the headers, the first 19 diagrams are for controls whereas the subsequent 28 plots depict tinnitus participants. The headers also indicate the maximum AAC (*max*) across the whole comodulogram. In each plot, the colour scale ranges from –*max* to *max* so that 0 is always coded by the same colour. Note that for each comodulogram *max* > |*min*| with *min* the minimum AAC so that the colour scale covers all assumed values.

More specifically, the maximum values range between 0.156 and 0.816 with a mean of 0.343, whereas minima range from –0.108 to –0.048 (m=–0.090). In each case, the majority of couplings is positive (proportions between 55.5% and 96.0%, m=71.5%).

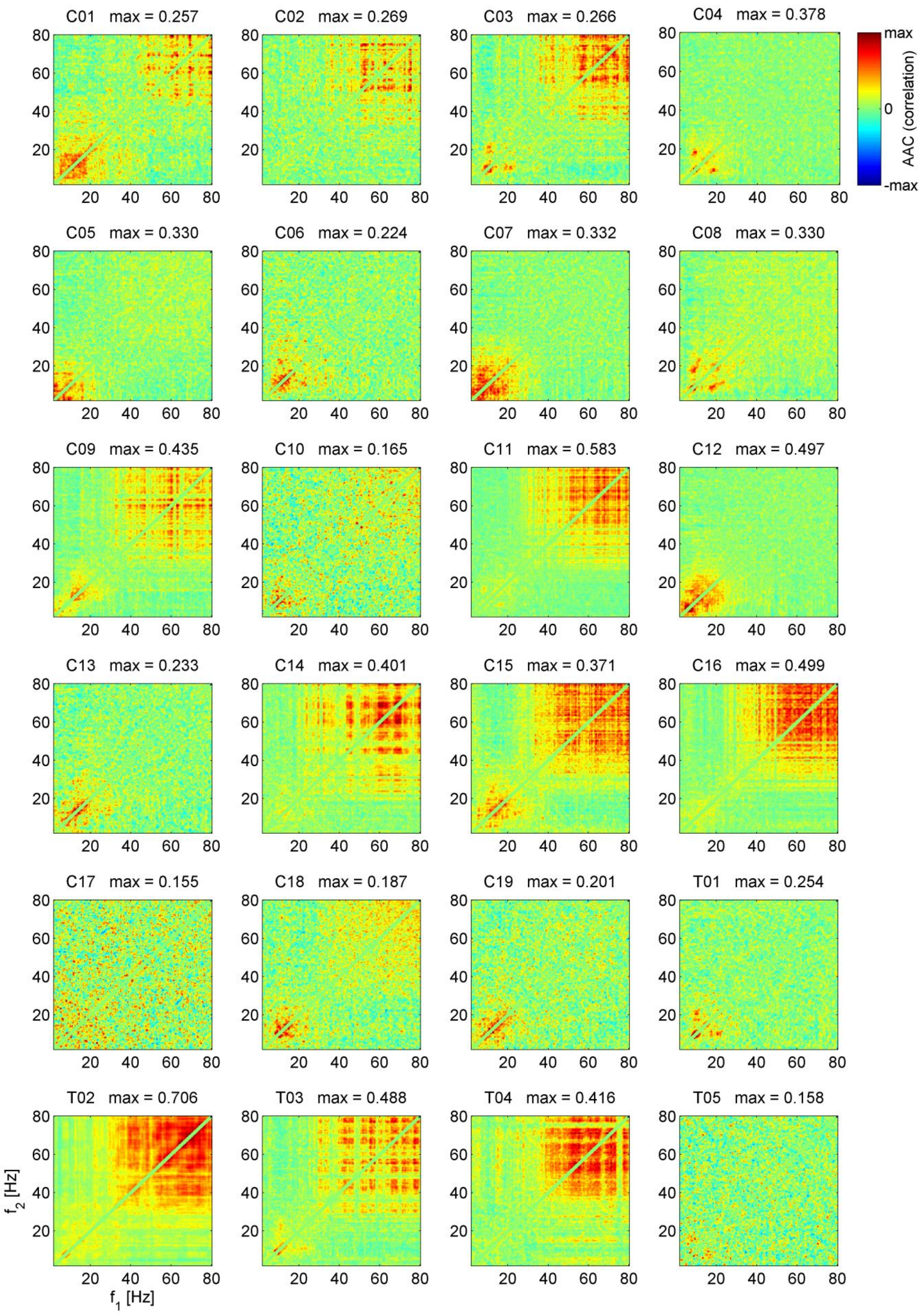
C01 max = 0.257
C02 max = 0.269
C03 max = 0.266
C04 max = 0.378
C05 max = 0.330
C06 max = 0.224
C07 max = 0.332
C08 max = 0.330
C09 max = 0.435
C10 max = 0.165
C11 max = 0.583
C12 max = 0.497
C13 max = 0.233
C14 max = 0.401
C15 max = 0.371
C16 max = 0.499
C17 max = 0.155
C18 max = 0.187
C19 max = 0.201
T01 max = 0.254
T02 max = 0.706
T03 max = 0.488
T04 max = 0.416
T05 max = 0.158
max
0
-max
AAC (correlation)
f_2 [Hz]
f_1 [Hz]

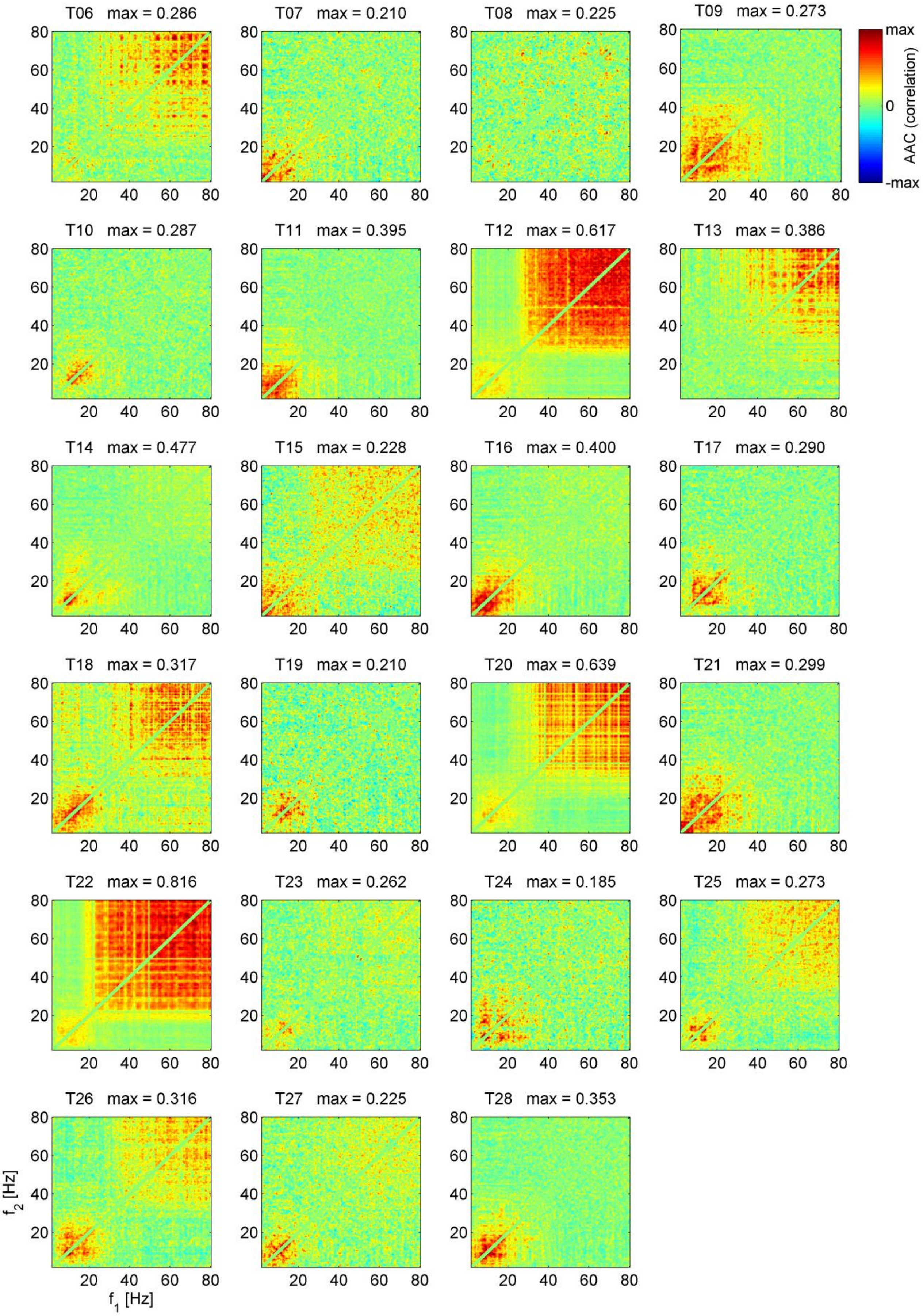
T06 max = 0.286
T07 max = 0.210
T08 max = 0.225
T09 max = 0.273
max
0
-max
AAC (correlation)
T10 max = 0.287
T11 max = 0.395
T12 max = 0.617
T13 max = 0.386
T14 max = 0.477
T15 max = 0.228
T16 max = 0.400
T17 max = 0.290
T18 max = 0.317
T19 max = 0.210
T20 max = 0.639
T21 max = 0.299
T22 max = 0.816
T23 max = 0.262
T24 max = 0.185
T25 max = 0.273
T26 max = 0.316
T27 max = 0.225
T28 max = 0.353
f2 [Hz]
f1 [Hz]

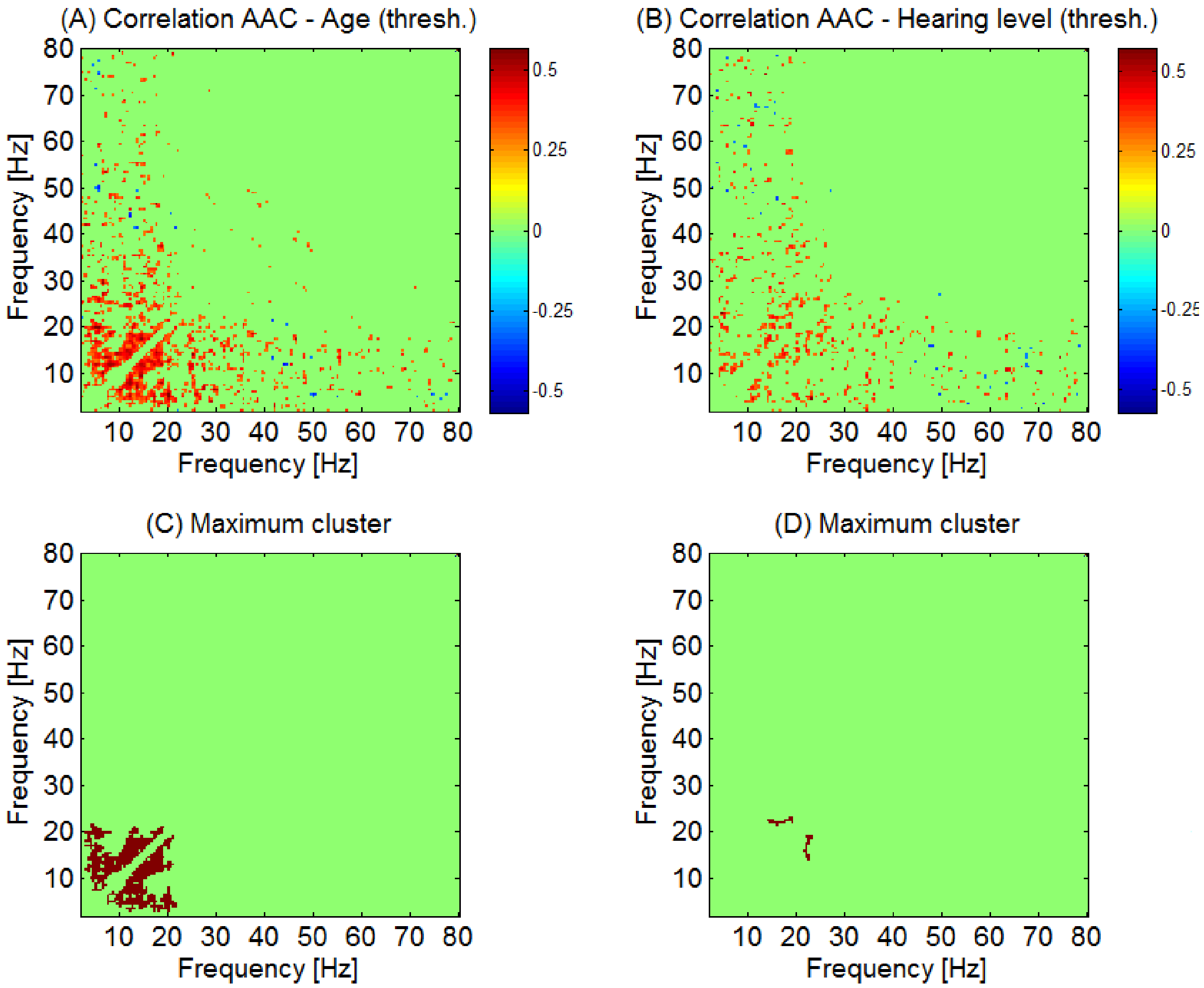

Figure S2. Correlation maps between AAC and (A) age and (B) hearing level thresholded at the cut-off levels for cluster definition ($|r| \geq 0.29$; see Methods section). The areas remaining after thresholding correspond to the observed clusters used in the cluster-based permutation tests. (C) and (D) show the respective clusters with the largest test statistics (i.e., correlation sums).

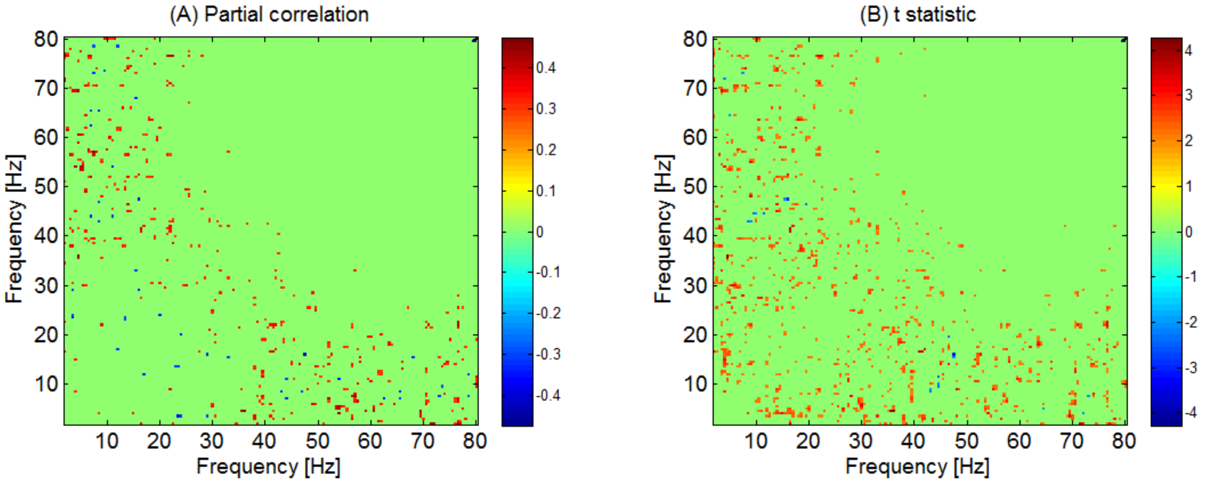

Figure S3. Thresholded maps of (A) partial correlations between AAC and group assignment controlling for age and (B) t statistics for the group comparison between tinnitus and control subjects.

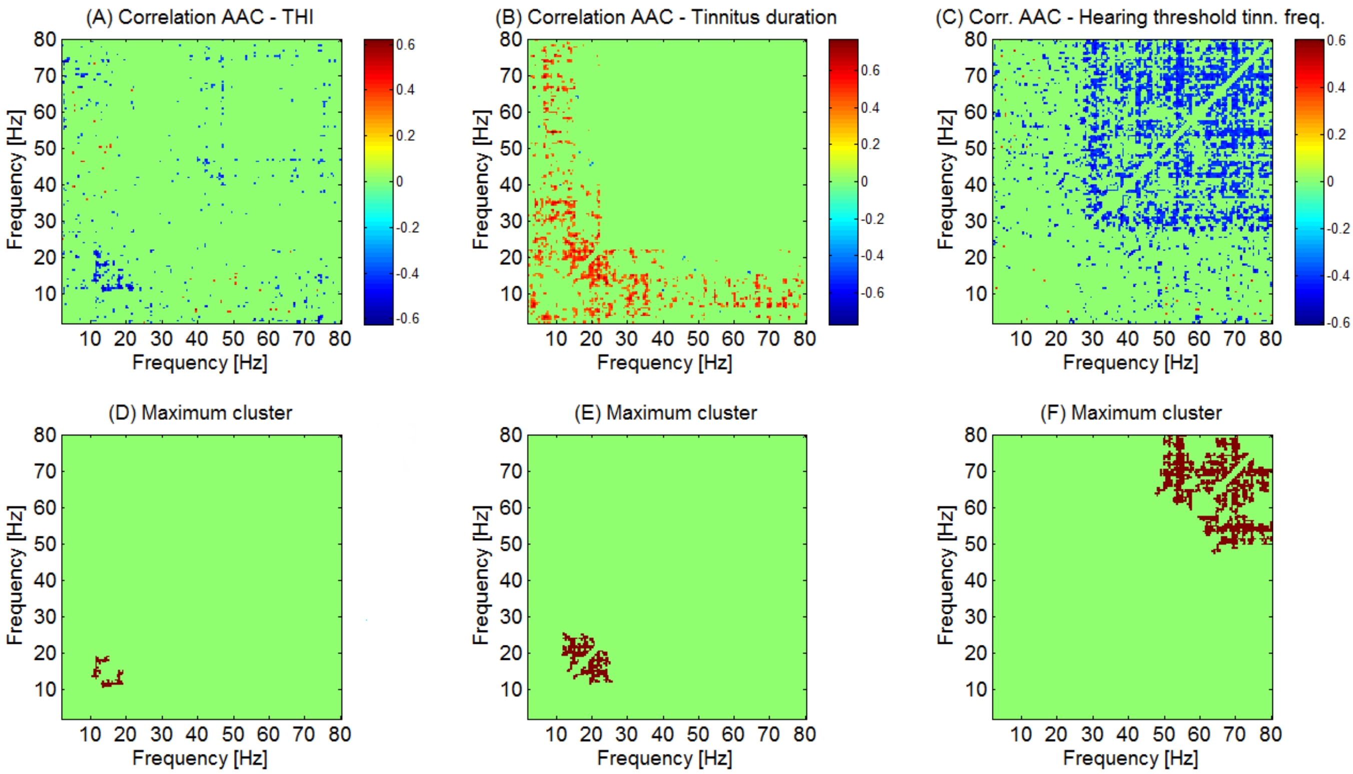

Figure S4. Thresholded correlation maps between AAC and (A) the THI score, (B) tinnitus duration and (C) the hearing threshold at the tinnitus frequency, showing the observed clusters used in the permutation tests. Figures (D)-(F) display the corresponding maximum clusters.

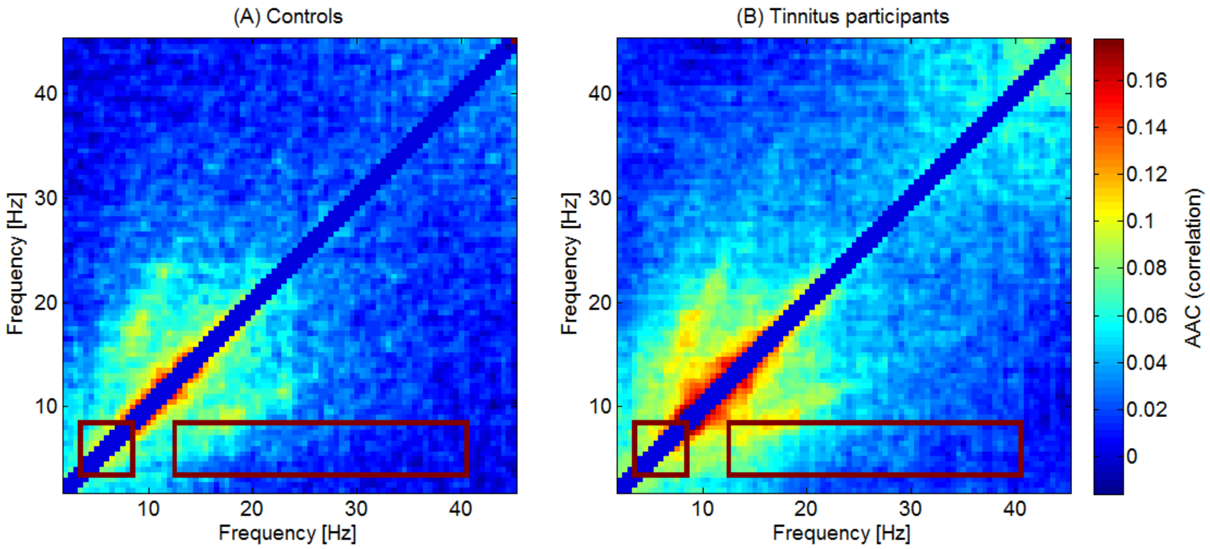

Figure S5. Subject-averaged comodulograms for (A) controls and (B) tinnitus participants. The diagrams highlight specific frequency windows of interest that were described in the TCD model of Llinas et al. [11,12].
